# Report of Cases in Ophthalmic Practice

**Published:** 1859-11

**Authors:** E. L. Holmes

**Affiliations:** Chicago


ARTICLE III.

### REPORT OF CASES IN OPHTHALMIC PRACTICE.

BY E. L. HOLMES, M.D., OF CHICAGO.

### CONGENITAL TUMOR ON THE CORNEA AND SCLEROTICA.

A young man, twenty-one years of age, in good health, called upon me regarding a singular congenital tumor, situated near the horizontal median line of the right eye, one half resting on the external portion of cornea and the other half on the sclerotica. The growth was of a yellowish white color, like a half-ripe cherry, oval in form, half an inch long, a quarter wide, and projecting three-eighths of an inch above the surface of the eye. When the lids were closed, the tumor passed under the upper lid, and raised it to such an extent from the lower that the edges of the lids were not in apposition. The tumor possessed so little sensibility, that it could be grasped firmly between the thumb and finger without producing pain, by which means it was found to be closely attached to the cornea, but movable over the sclerotica. There was a slight strabismus, convergent with so much dimness of vision, that the eye was almost entirely useless. Still the patient experienced scarcely any inconvenience from the tumor, since he had become accustomed to its existence, and enjoyed excellent vision in the other eye. The aspect of the eye was very unpleasant, for which reason alone the patient wished the tumor removed.

The operation was exceedingly simple. After chloroform had been administered, the tumor was seized with a pair of small-toothed forceps as near the globe as possible, and removed by paring it from its seat with a small bistoury, leaving the walls of the eye of their normal thickness. Although the sclerotical portion of the tumor appeared somewhat movable, it was found impossible to *dissect* it from its bed. It was pared from the sclerotica as from the cornea, care being taken to leave the surface of the whole wound convex—uniform with that of the eye.

The wound healed, with scarcely a symptom of inflammation; —not a trace of it appeared on the healthy portion of the cornea. A few granulations formed in the centre of the wound, which were wholly removed by two or three applications of nitrate of silver. In three weeks cicatrization was complete, the two lateral hemispheres of the eye being perfectly symmetrical in form. As might have been anticipated, the cornea presented a dense opacity at the seat of the tumor; the portion of the cicatrix upon the sclerotica, however, is much less perceptible.

Although the operation was performed principally to satisfy the vanity of the patient, it has proved to be of decided advantage to him. The strabismus has entirely disappeared, and vision in this eye has been constantly improving since the removal of the tumor,—a result, doubtless, to be explained by the fact, that images of objects are now formed in both eyes on corresponding portions of the retinæ.

The tumor, which resembled, in some respects, the small growths occasionally seen upon the sclerotica (pinguicula), was probably a lipoma, a full description of which can be found in the works of Desmarres and other authors.

I am indebted to Drs. Freer and Hunt for a microscopical examination of the tumor, which they found to be of a fibrous and fatty structure.

### ACUTE IRITIS, WITH EXTRAVASATION OF BLOOD INTO THE INTERIOR CHAMBER.

A gentleman, about fifty years of age, was attacked with inflammation of the right eye. It was not till the fourth day that he was unable to attend to his usual business, when he sought the advice of his physician, Dr. Max Myers, of this city, who found the case of some interest, and kindly favored me with an opportunity of observing the disease through its course.

At my first visit (May 3), the eye presented the following appearances: the lids were considerably swollen; the conjunctiva of the globe extensively chemosed; the iris discolored and apparently œdematous; the pupil contracted and irregular; the aqueous humor quite turbid. The subjective symptoms were photophobia, dimness of vision, with pain in and around the eye, which last symptom, however, was much less severe than in similar cases that I have seen. The patient had already taken a full dose of pil. hyd., and had been subjected to a rigid diet; extract belladonna and warm fomentations of poppy leaves had been freely applied around the eye. It was, therefore, thought best simply to scarify the chemosed conjunctiva and substitute a solution of sulphate of atropine (gr. ij. aquæ ℥ ss.) for the belladonna.

The following morning (May 4) a singular and somewhat alarming symptom was observed. During the night, evidently while the patient was sleeping upon his right side, blood was extravasated from the vessels of the iris in such quantities as to fill the temporal portion of the anterior chamber as far as the outer edge of the pupil. In this position it had coagulated and become jet black. In the erect position of the head, this coagulum, instead of settling to the lower portion of the anterior chamber, remained perpendicular. Pus in the anterior chamber (hypopium), as is well known, usually changes its position in the beginning, according to the direction in which the head is held. Although the chemosis had subsided, the eye was, in other respects, in a worse condition than before. The aqueous humor was more turbid; the iris apparently more discolored; vision almost completely destroyed, although the pupil was somewhat dilated; the globe presented that peculiar dark, livid red appearance so often seen in similar cases. The pain was not very severe.

It was thought best to subject the patient more decidedly to the influence of mercury. For this purpose a grain of calomel and an eighth of a grain of opium were given every hour; two leeches were applied to the side of the nose near the angle of the eye. The atropine was continued as previously directed. In the evening I saw the patient again, and found he had been sitting up all day. The lower portion of the anterior chamber was filled with black coagulated blood almost to the centre of the pupil.

The aspect of the eye was certainly peculiar. Full two-thirds of the anterior chamber was filled with these jet-black coagula, the level surface of the second forming a right angle, of course, with the perpendicular surface of the first. The prognosis, as regarded vision, was exceedingly unpromising. No change was made in the treatment except to order a little morphine, if the patient should need it to quiet pain or induce sleep.

The next day (May 5) the symptoms were more favorable. The patient had rested well; the pain was less; and the extravasation had not increased. The calomel was ordered to be taken every three hours when awake. During the three following days, there was a wonderful improvement in the condition of the eye. It was becoming less red and painful, and the process of absorption was rapidly taking place. The calomel was now given only three times daily. Continued treatment in other respects as before. On May 13, patient was able to resume, to a certain extent, his business, although apprized of the danger resulting from exposure. At this time the coagula had entirely disappeared; the conjunctiva was only slightly congested; there was no pain. The dimness of vision, of which the patient complained, was probably caused by the dilatation of the pupil and by the turbid condition of the aqueous humor.

From this time there was a constant improvement in the appearance of the eye and in vision. The pupil resumed its normal size and movements, as soon as the atropine was omitted. In a few weeks the patient was able to perform all the duties of his profession without the least difficulty. The cure was perfect.

The case is of peculiar interest, not only as showing to what extent the eye can suffer from inflammation and yet recover, but also as being an example of extensive extravasation of blood into the anterior chamber in iritis. A small quantity of blood *with* pus is not unusual in this disease; but such large coagula without the least apparent trace of pus is quite rare.

(TO BE CONTINUED).

